# Evidence-Based Periodic Health Examinations for Adults: A Practical Guide

**DOI:** 10.7759/cureus.79963

**Published:** 2025-03-03

**Authors:** Gabriel C Araujo, Caio B Ribeiro, Maria Clara M Costa, Maria Luisa P Evangelista, Mariana F Lima, Mateus C De Paula, Vitoria L Ferreira, Fernando Antonio Glasner da R Araujo

**Affiliations:** 1 Internal Medicine, Orion - Orientacao Medica, Salvador, BRA; 2 Internal Medicine, Universidade Federal da Bahia, Salvador, BRA; 3 Internal Medicine, Escola Bahiana de Medicina, Salvador, BRA; 4 Internal Medicine and Diagnostics, Universidade Federal da Bahia, Salvador, BRA

**Keywords:** annual health check, checkup, clinical checkup, evidence-based clinical guidelines, evidence-based clinical practice, overdiagnosis, periodic health examination, preventive medicine, screening

## Abstract

Periodic health examinations, or annual clinical checkups, are a primary reason for seeking medical care. The objective is to identify hidden problems or diseases in their early stages and to promote behaviors that prevent or minimize the consequences of these conditions. However, the practice of conducting annual reviews with clinical, laboratory, and imaging examinations indiscriminately is not associated with outcomes that matter to the patient (such as reducing morbidity and mortality) and may result in harm, including overdiagnosis and overtreatment. The objective of the present work is to review and summarize the measures currently recommended and supported by scientific evidence from the main regulatory authorities of the United States (United States Preventive Services Task Force (USPSTF)) and Canada (Canadian Task Force on Preventive Health Care (CTFPHC)), in order to create a practical guide for evidence-based checkups.

## Introduction and background

One of the main reasons for medical visits is to conduct periodic evaluations in asymptomatic individuals, also known as clinical checkups, periodic health exams, or annual reviews. In the United States, in 2020, this was the primary reason for medical care, accounting for 30.5% of all consultations in community health centers. Following this, seeking care for a complaint (29.2%) and follow-up for a previous illness (17.3%) were the next most common reasons [1].

The idea that periodic examinations could uncover hidden problems, leading to early curative intervention, seems to have originated at least two centuries ago, with health insurers requiring medical examinations to reduce their financial risk by identifying hidden clinical issues [2]. Despite the passage of time, the belief that discovering problems early is always advantageous remains strong among the general population and even among physicians. However, "early diagnosis," often described as essential to "avoid" unfavorable outcomes, carries risks and potential harm.

An important concept to keep in mind when performing tests is avoiding overdiagnosis. Overdiagnosis refers to the detection of a condition that would not cause symptoms or harm to the patient during their lifetime. This occurs when diagnostic tests identify diseases or abnormalities that progress slowly, would never evolve into a significant threat, or could even regress spontaneously. Additionally, overdiagnosis can lead to unnecessary treatments, adverse effects, and anxiety in patients, without providing real health benefits [3].

One of the reasons behind the false impression of the importance of early diagnosis is lead time bias. This type of bias occurs in screening tests when the early detection of a disease creates the false impression that patient survival has increased, even though there has been no real improvement in clinical outcomes. In other words, patients who undergo screening are diagnosed with the disease earlier; however, if their age of death is no different from those who did not undergo screening and discovered the disease shortly before they died, early detection did not change the patient's life expectancy [4].

Overuse and inappropriate use of screening tests are the primary causes of overdiagnosis [5]. Therefore, it is essential for physicians to guide their practice based on evidence-based recommendations, especially when dealing with asymptomatic patients, who are not natural candidates for medical interventions.

The first official recommendation from a scientific entity that we were able to identify was made by the American Medical Association (AMA) in 1922. They recommended, in addition to a detailed anamnesis and physical examination, the opportunistic use of hygiene advice, especially those related to diet, physical exercise, oral and personal hygiene, and sleep [6].

In 1976, the Canadian Task Force on the Periodic Health Examination (now the Canadian Task Force on Preventive Health Care - CTFPHC) was created to "determine how periodic health examinations can improve or protect the health of the population in Canada," which resulted in the publication of its first set of recommendations three years later [7]. These recommendations were the first to be based on scientific evidence and graded according to the quality of the evidence. Similarly, in 1998, the US Preventive Services Task Force (USPSTF) published its first Clinical Guide to Preventive Services, featuring 169 interventions related to 60 clinical conditions [8]. Since then, both the CTFPHC and the USPSTF have periodically updated their recommendations.

Both institutions base their recommendations on rigorous, multidisciplinary, and periodic analyses of scientific evidence, grading their guidelines according to the strength and methodological quality of this evidence. The USPSTF classifies the conducts as "recommended" (A and B, depending on the strength of the evidence), "recommended for special cases" (C), "insufficient data for recommendation (I), up to "recommendation against" the evaluated conduct (D). Although they cannot be directly compared, the CTFPHC also classifies its guidelines by virtue of recommendation. "Strong recommendation" is one for which there is a greater probability of certainty that the desirable effects of an intervention outweigh its undesirable effects or that the undesirable effects of an intervention outweigh its desirable effects. Conditionals are those for which desirable effects are likely to outweigh undesirable effects or undesirable effects are likely to outweigh desirable effects, but uncertainty exists. Conditional recommendations result when the balance between desirable and undesirable effects is small, the quality of the evidence is lower, and there is more variability in individuals' values and preferences.

Although several recommendations regarding periodic health screenings are made by different scientific societies in various countries, the authors chose to focus on the recommendations of the USPSTF and CTFPHC, as they have a broader scope (most others are limited to specific cancer screening recommendations), a rigorous methodology, and more universal acceptance.

A fundamental concept is that these measures are not recommended as the classic "annual checkup," but rather as periodic preventive health measures [9]. The frequency varies for each of them, ranging from only once in the patient’s lifetime to being repeated in every doctor-patient interaction.

The objective of this study is to review the recommendations made by the North American (grades A and B) and Canadian (strong or conditional) task forces, consolidating and summarizing a routine for periodic health assessment of non-pregnant adults, based on updated evidence.

## Review

Material and methods

The most up-to-date versions of the CTFPHC [10] and USPSTF [11] recommendations were reviewed by consulting the websites of both entities.

The following inclusion criteria were established: (1) the most recently published active recommendation; (2) "in favor" recommendations from both the USPSTF and CTFPHC; (3) "in favor" recommendations from one institution when there is no corresponding recommendation from the other, or when there is disagreement (explicitly stated in the text); and (4) recommendations limited to non-pregnant adults.

It is important to note that by choosing not to include conditional recommendations from the USPSTF (grade C), we excluded some notable recommendations, such as prostate cancer screening.

Results

We describe below the screening and counseling measures currently recommended by USPSTF and the CTFPHC. Some of these measures, at the time of writing this review (January 2025), were in the process of being updated.

Recommendations for Adults in General

Tobacco smoking cessation in adults: The USPSTF recommends screening all adults for smoking (grade A). This screening should be repeated at every medical consultation, "as if it were a vital sign" [12]. In the case of a positive screening result, the patient should be advised to quit smoking and undergo behavioral and pharmacological interventions. If a specialized smoking cessation service is available, the patient should be referred to it.

Screening and behavioral counseling interventions for unhealthy alcohol use in adolescents and adults: The USPSTF recommends screening for unhealthy alcohol use in adults 18 years and older (grade B) [13]. Accurate screening instruments are available to identify harmful alcohol use, such as the Alcohol Use Disorders Identification Test-Consumption (AUDIT-C) [14] and the Single Alcohol Screening Question (SASQ) [15]. Evidence suggests that while the CAGE questionnaire is widely used in clinical practice, it only detects alcohol dependence and is not useful for assessing other disorders associated with harmful alcohol use. Once an increased risk of harmful use is identified, the patient should be informed, counseled, and referred for a more specialized diagnostic and therapeutic approach. There is insufficient evidence to determine an appropriate interval for repeat screening.

Unhealthy drug use: The USPSTF recommends screening for unhealthy drug use in adults (grade B) [16]. This recommendation applies to the use of illicit drugs and does not cover alcohol, tobacco, or prescription substances. Screening should be conducted through direct questioning about drug use during the interview and does not involve toxicological examination of biological samples. A validated instrument for screening is the National Institute on Drug Abuse (NIDA) questionnaire, available in its Quick Screen or Full Form (ASSIST) [17]. There is insufficient evidence to determine an appropriate interval for repeat screening. As with alcohol use, once a risk of harmful drug use is identified, the patient should be informed, counseled, and referred for a more specialized approach.

Anxiety disorders in adults: The USPSTF recommends screening for anxiety disorders in adults up to age 64, including pregnant and postpartum women (grade B) [18]. This recommendation applies to the general adult population, but it is especially important for those with a family history, other mental disorders, a history of stressful events, smoking or alcohol consumption, or those who are divorced or widowed. Evidence on the optimal timing for screening or the need for periodic repetition is limited. A pragmatic approach would be to screen all adults who have not been previously screened and use clinical judgment to consider risk factors, clinical conditions, and life events to determine whether additional screening is needed. Selected screening instruments widely used in the US include versions of the Generalized Anxiety Disorder (GAD) scale [19], the anxiety subscale of the Edinburgh Postpartum Depression Scale (EPDS) [20], and the Geriatric Anxiety Scale (GAS) [21]. These instruments are not intended for the diagnosis of anxiety disorders. If a screening test result is positive, patients should be referred for a confirmatory diagnostic evaluation and evidence-based care.

Depression and suicide risk in adults: The USPSTF recommends screening for depression in adults (grade B) [22]. Screening instruments previously validated and recommended by the USPSTF include the Patient Health Questionnaire (PHQ-2) [23], the Center for Epidemiologic Studies Depression Scale (CES-D) [24], and the Geriatric Depression Scale (GDS) [25]. If the screening is positive, the patient should be referred to a professional who can properly diagnose and treat depression. The CTFPHC, on the other hand, recommends against screening for depression in adults (weak recommendation, very low-certainty evidence). The CTFPHC emphasizes that while evidence of benefit is limited, the systematic review of the current guideline did not identify any studies evaluating the harms of screening. Potential harms include false-positive diagnoses leading to unnecessary treatment, adverse effects of medical therapy in people correctly identified as having depression, and the consequences of labeling and stigma. However, while recommending against screening, the CTFPHC urges clinicians to remain vigilant for signs of depression, particularly in patients with insomnia, depressed mood, anhedonia, and suicidal thoughts [26].

Hypertension in adults: Both the USPSTF and the CTFPHC recommend screening for systemic arterial hypertension in individuals over 18 years of age, without a previous history of hypertension (grade A/strong) [27,28]. Pressure should be measured by a manual or automated sphygmomanometer, in the brachial artery, with the patient seated and after five minutes of rest. The screening interval suggested by the USPSTF is annual for people over 40 years of age and for those who are at an increased risk of hypertension. Screening can be done less frequently, that is, every three to five years, for people between 18 and 39 years old, who do not have an increased risk and with previous blood pressure measurement without changes. The CTFPHC, on the other hand, recommends screening in all consultations with new patients, periodic exams, neurological or cardiovascular emergency care, medication renewal consultations, and other occasions in which the doctor considers it appropriate to monitor blood pressure. The frequency and timing of screening may vary according to age, comorbidities, and the presence of other cardiovascular risk factors. If the screening is positive, the USPSTF recommends obtaining blood pressure measurements outside the office, through ambulatory blood pressure monitoring (ABPM) and home blood pressure monitoring (HBPM) for eventual diagnostic confirmation and initiation of treatment. The CTFPHC indicates that patients who have characteristics of hypertensive urgency or emergency can already be diagnosed based on this initial evaluation. The others need additional measurements in subsequent consultations.

Laboratory and Other Tests

Prediabetes and type 2 diabetes: The USPSTF recommends (grade B) screening for prediabetes and type 2 diabetes in asymptomatic adults aged 35 to 70 years who are overweight or obese [29]. Clinicians should consider screening at an earlier age in individuals with a family history of diabetes, a prior diagnosis of gestational diabetes or polycystic ovarian syndrome, or those who are part of groups with disproportionately high incidence and prevalence (American Indian/Alaska Native, Asian American, Black, Hispanic/Latino, or Native Hawaiian/Pacific Islander) [29].

Although the CTFPHC also recommends screening in individuals at "high risk" or "very high risk" (as determined by the Finnish Diabetes Risk Score (FINDRISC) score, or alternatively by the Canadian Diabetes Risk Questionnaire (CANRISK)), the evidence was rated as weak and supported by low-quality evidence. The CTFPHC recommends screening with HbA1c every three years (if previous testing was normal) for "high-risk" patients and annually for those at "very high risk" [30].

According to the USPSTF, prediabetes and type 2 diabetes can be detected by measuring fasting blood glucose, HbA1c, or blood glucose levels two hours after an overload. A fasting plasma glucose level greater than or equal to 126 mg/dL (6.99 mmol/L), an HbA1c greater than or equal to 6.5%, or a blood glucose level two hours after overload greater than or equal to 200 mg/dL (11.1 mmol/L) is consistent with the diagnosis of type 2 diabetes. A fasting plasma glucose level between 100 and 125 mg/dL (5.55-6.94 mmol/L), an HbA1c between 5.7% and 6.4%, or a two-hour post-load blood glucose level between 140 and 199 mg/dL (7.77-11.04 mmol/L) is consistent with prediabetes.

Evidence on the optimal screening interval for adults with an initial normal glucose test result is limited. Cohort and simulation model studies suggest that screening every three years may be a reasonable approach for adults with normal blood glucose levels. Clinicians should offer or refer patients with prediabetes to effective preventive behavioral interventions and consider the use of metformin. Some studies suggest that metformin is effective in individuals younger than 60 years, those with a BMI of 35 or more, those with a history of gestational diabetes, and those with a fasting plasma glucose level of 110 mg/dL (6.11 mmol/L) or higher.

Human immunodeficiency virus infection: The USPSTF recommends testing for human immunodeficiency virus (HIV) infection in adolescents and adults aged 15 to 65 years (grade A) [31]. Younger adolescents and older adults at higher risk of infection should also be screened. Individuals at higher risk for HIV infection include men who have sex with men, people with multiple sexual partners with whom they engage in unprotected vaginal or anal intercourse, individuals who exchange sex for drugs or money, injecting drug users, those seeking treatment for other sexually transmitted infections or who have infected partners, people with a history of blood transfusions between 1978 and 1985, transgender individuals, and those who request HIV testing even without explicit risk factors.

The testing algorithm introduced by the CDC in 2014 begins with a combined assay that detects antibodies against HIV-1 and HIV-2, as well as HIV-1 p24 antigens. Supplemental tests are performed after reactive results to differentiate between HIV-1 and HIV-2 antibodies. In cases of non-reactive or indeterminate supplemental testing, an HIV-1 RNA test is used to distinguish between an acute HIV infection and a false-positive result.

Current evidence is insufficient to establish optimal time intervals or strategies for repeat HIV screening. However, it is reasonable to repeat testing in individuals who remain at high risk of infection, as well as in those who live in or receive medical care from high-prevalence settings, such as STD clinics, tuberculosis clinics, correctional facilities, or homeless shelters.

Hepatitis C virus infection: The USPSTF recommends screening for HCV infection in adults aged 18 to 79 years (grade B). The test should be done only once for the general population. In at-risk populations, the test should be repeated periodically as long as the risk persists [32]. The CTFPHC, however, only recommends screening for individuals at risk of HCV infection (strong recommendation, very low-quality evidence) and recommends against general population screening [33]. This difference in recommendations is explained by the CTFPHC, which cites the possibility of false-positive tests (especially serological tests) leading to anxiety or unnecessary treatments, as well as the cost-benefit ratio of testing the entire population. However, both agree on periodic screening (without a defined interval) for at-risk populations. In this regard, the USPSTF is more restrictive, defining people at risk as those who use injectable drugs or engage in risky sexual behavior. The CTFPHC is more comprehensive, expanding the group of those screened to include individuals who have used or currently use injectable drugs; have been incarcerated; were born, traveled, or resided in countries with HCV endemicity; received medical care in countries with limited universal precautions; received blood transfusions or organ transplants prior to 1992 in Canada; are on hemodialysis; have suffered needlestick injuries; engage in or have engaged in risky sexual behavior; are homeless; have used intranasal or inhalation drugs; have tattoos or piercings; and share sharp personal hygiene instruments with HCV-positive individuals or those showing other clinical signs of suspected HCV. Screening should be conducted with an anti-HCV serological test, followed by confirmation using HCV RNA for positive cases.

Hepatitis B virus infection: Screening for HBV infection is recommended for all adolescents and nonpregnant adults at increased risk (grade B) [34]. Individuals at increased risk include those born in countries and regions with a high prevalence of HBV infection (≥2%), such as Asia, Africa, the Pacific Islands, and parts of South America, unvaccinated individuals, HIV-positive individuals, injecting drug users, men who have sex with men, and household contacts or sexual partners of people with HBV infection.

Screening should be done using an HBsAg test, followed by a confirmatory test in case of reactive results. Patients with a negative HBsAg test result who have not been fully vaccinated against the disease and who remain at risk of HBV transmission should be tested periodically. However, there is no sufficient evidence to determine an optimal testing interval.

Syphilis infection in nonpregnant adolescents and adults: Screening for syphilis infection is recommended for adults who have risk factors for this condition (grade A) [35]. High-risk groups include men who have sex with men, people living with HIV, and those in communities with a high prevalence of the infection.

There are two main approaches to testing. The first is the traditional algorithm, which starts with a non-treponemal test, and if the result is positive, it is confirmed with a treponemal antibody detection test. The second approach is the reverse sequence algorithm, which begins with an automated treponemal test, and if positive, is confirmed with a non-treponemal test. Discordant results in the reverse sequence are resolved with a second confirmatory treponemal test, preferably one that detects different antigens than the initial test.

Confirmed cases should be treated accordingly and provided with guidance on prevention measures. Regarding screening intervals, it is recommended that men who have sex with men and HIV-infected individuals be screened at least once a year. For those who remain at high risk of infection, the screening interval may be shortened to every three to six months.

Latent tuberculosis infection in adults: Screening for LTBI (latent tuberculosis infection) is recommended for adults within populations at increased risk (grade B) [36]. At-risk populations include individuals born in, residents of, or former residents of countries with a high prevalence of tuberculosis, as well as residents of congregate settings (such as shelters or prisons), immunosuppressed individuals, people with silicosis, contacts of individuals with active tuberculosis, and healthcare workers.

Screening can be done with a tuberculin skin test or PPD (purified protein derivative) test, or with an interferon-gamma release assay (IGRA). If IGRA is available, it is preferable, as it is faster, does not require a follow-up reading, and is not affected by the BCG vaccine. The evidence regarding the need for periodic testing remains inconclusive. Evidence suggests single testing for patients at lower risk, with annual retesting for those at high risk or with continuous exposure. In patients with positive test results, additional tests are necessary to diagnose active tuberculosis.

Colorectal cancer: Both entities recommend screening for colorectal cancer in adults, differing in age and type of screening method. The USPSTF recommends screening for all adults aged 50-75 (grade A). For those aged 45-49, screening should be offered to individuals who wish to undergo it, regardless of whether they are from risk populations, and it is indicated for those in high-risk groups (men, African Americans, those with a family history of colorectal cancer, obesity, diabetes, long-term smoking, or alcohol abuse) (grade B) [37]. The CTFPHC recommends screening for all individuals aged 60-74 (strong recommendation) [38].

The most commonly used screening tests can be divided into two types: tests performed on stool samples and tests involving direct visualization. Among the stool sample tests are the fecal occult blood test, the fecal immunochemical test (FIT), and the fecal DNA test with fecal immunochemical test (sDNA-FIT). The most commonly used direct visualization tests include colonoscopy and flexible sigmoidoscopy.

The CTFPHC recommends stool testing (guaiac or FIT) every two years, or flexible sigmoidoscopy every 10 years [38]. The USPSTF allows screening with any of the available methods; however, it considers colonoscopy, performed every 10 years, to be the gold standard [37]. The recommended testing intervals in both entities refer to previous tests with no significant findings. Once abnormalities are detected, the interval or subsequent examinations should be guided by professionals trained in these situations.

Lung cancer: The USPSTF recommends that adults aged 50 to 80 years with a smoking history of 20 pack-years or a history of smoking and less than 15 years of abstinence undergo annual screening with low-dose computed tomography (grade B) [39]. The CTFPHC has a similar recommendation, with differences in age and smoking burden, but considers the evidence to be weak and of low quality [40]. Screening should be conducted annually if the established conditions persist. Screening should be discontinued when the patient has completed 15 years of abstinence or develops a health condition that substantially limits life expectancy or the ability or willingness to undergo curative lung surgery.

Counseling

Promotion of a healthy diet and exercise: The USPSTF recommends that adults with cardiovascular risk factors be advised to maintain a healthy diet and engage in regular physical activity (grade B) [41]. An adult with cardiovascular risk factors is defined as an individual aged 18 years or older who has one or more of the following factors: hypertension, dyslipidemia, metabolic syndrome, or an estimated 10-year cardiovascular risk of 7.5% or greater, calculated using the Pooled Cohort Equations or the Framingham Risk Score [42].

To be effective, the orientation must be intensive, involving multiple contacts (approximately 30 minutes per month), with individual or group counseling sessions over a long period (one year). Dietary advice includes reducing the consumption of saturated fats, sodium, and sweets/sugars, and increasing the intake of fruits, vegetables, and whole grains. Regarding physical activity, the individual should aim for 90 to 180 minutes per week of moderate to vigorous activity, respectively.

Ideally, the program should be led by an interventionist with knowledge of motivational techniques, behavior change, goal setting, active self-monitoring, and addressing barriers related to diet, physical activity, or weight change. Non-medical professionals, such as nurses, nutritionists, exercise specialists, and physiotherapists, should also participate.

Weight loss in obese individuals: The USPSTF recommends that adults with a body mass index (BMI) of 30 or greater undergo intensive, multicomponent behavioral interventions for weight reduction (grade B) [43]. The individual should be provided orientation or referred for specialized guidance regarding behavioral changes, including dietary modifications, physical activity, identification of barriers to healthy habits, self-monitoring of weight, support from friends or family, use of support tools (such as pedometers, food intake trackers, and exercise videos), and strategies to prevent weight regain.

Sexual behavior: The USPSTF recommends behavioral counseling for all sexually active adolescents and adults at increased risk of sexually transmitted infections (STIs) (grade B) [44]. The recommendation defines STIs as those resulting from sexual activity or intimate physical contact and cites the most common STIs in the USA as HIV, herpes simplex virus, human papillomavirus (HPV), hepatitis B virus (HBV), Chlamydia trachomatis, Neisseria gonorrhoeae, Treponema pallidum (syphilis), and Trichomonas vaginalis.

People at risk of STIs are those with a current or recent STI (within the past year), those who do not use condoms consistently, have multiple sexual partners, or have sexual partners in populations with a high prevalence of STIs (such as individuals who test or attend STI clinics, sexual and gender minorities, people living with HIV, those who inject drugs, exchange sex for money or drugs, or have been incarcerated). The clinician should provide counseling on safe sex practices and inform patients about the symptoms and transmission methods of common STIs. When available, the clinician should refer the patient to specialized services and provide educational materials (e.g., leaflets and videos).

Skin cancer prevention: The USPSTF recommends minimizing exposure to UV radiation for individuals up to 24 years of age with fair skin type (light hair and eyes, freckles, and those who burn easily in the sun) (grade B) [45]. Although it is a grade C (conditional) recommendation, individuals over 24 years of age with a personal or family history of skin cancer, a high number of nevi (moles), atypical nevi, HIV infection, or a history of receiving an organ transplant may also benefit from protection. Suggested protective measures include avoiding sun exposure, using a broad-spectrum sunscreen with a sun protection factor (SPF) of 15 or higher, wearing hats, sunglasses, and sun-protective clothing, seeking shade during peak sun hours (10 a.m. to 4 p.m.), and avoiding artificial tanning.

Fall prevention in community-dwelling older adults: The USPSTF recommends exercise interventions to prevent falls in community-dwelling adults aged 65 and older who are at increased risk for falls (grade B) [46]. Important warning signs for fall risk include a history of previous falls, as well as problems with mobility and balance. Other risks to consider are advanced age, cognitive and sensory deficits, the presence of acute or chronic medical conditions, the use of medications that may increase the risk of falling, environmental or occupational hazards, household or neighborhood characteristics, and alcohol or drug use.

Initially, behavioral, environmental, and medical factors must be considered and addressed. Next, the patient should be encouraged to engage in physical activity, emphasizing its multiple health benefits in addition to fall prevention, including reduced mortality and a lower risk of type 2 diabetes and dyslipidemia. The most studied physical activities include gait, balance, and functional training, followed by strength and endurance training, flexibility, and resistance training. Treatment should be multidisciplinary, involving physical activity professionals and, if necessary, geriatricians.

Prevention of HIV acquisition through pre-exposure prophylaxis: The USPSTF recommends that physicians prescribe pre-exposure prophylaxis, using effective antiretroviral therapy, for individuals at increased risk of acquiring HIV to reduce this risk (grade A) [47]. Individuals covered by this recommendation include sexually active adults and adolescents who have engaged in anal or vaginal sex in the past 6 months and have any of the following: a sexual partner with HIV; a sexually transmitted bacterial infection (STI) within the past 6 months; or a history of inconsistent or nonexistent condom use with sexual partner(s) whose HIV status is unknown. Regarding the last point, the risk should be assessed in conversation with the patient, considering factors such as the number of partners, the specific sexual activities a person engages in, or the group of sexual partners with a higher prevalence of HIV (e.g., men who have sex with men or with both men and women, transgender women, people who inject drugs, and people who exchange sex for payment).

The recommended drugs may vary based on the epidemiological or legal conditions of each country, as well as the possible mode of transmission (anal/vaginal sex, injection drug use). These measures apply only to individuals who remain HIV-negative. They should be advised to adhere to prescribed medications and safe sex practices, including condom use, regular HIV testing, and other preventive measures.

Statin use for the primary prevention of cardiovascular disease in adults: The USPSTF recommends that physicians prescribe a statin for the primary prevention of cardiovascular disease (CVD) in adults aged 40 to 75 who have one or more CVD risk factors (dyslipidemia, diabetes, hypertension, or smoking) and an estimated 10-year risk of a cardiovascular event of 10% or greater (grade B) [48]. The higher the 10-year risk of cardiovascular events, the greater the potential benefit from statin use. However, data on the recommended statin dose is limited. These recommendations do not apply to adults with an LDL cholesterol level greater than 190 mg/dL (4.92 mmol/L) or known familial hypercholesterolemia, as specific guidelines from other organizations are recommended for these very high-risk CVD populations.

Specific Recommendations for Adult Females

Chlamydia and gonorrhea screening: The USPSTF recommends screening for chlamydia and gonorrhea in all sexually active women up to the age of 24, and in women aged 25 and older who are at increased risk of infection (grade B) [49]. Although the CTFPHC considers the evidence weak and of low or very low certainty, it views screening as a recommendation that could reduce the incidence of pelvic inflammatory disease in women, even if the benefits are uncertain. It is considered a conditional recommendation, particularly for women aged 15 to 29 years [50]. Those at increased risk include individuals with a personal or partner history of STIs, a new sexual partner or multiple partners, a partner who has sex with others, inconsistent condom use outside of a monogamous relationship, a history of exchanging sex for money or drugs, or a history of incarceration.

The recommended test is the nucleic acid amplification test (NAAT) for Neisseria gonorrhoeae and Chlamydia trachomatis. In addition to its excellent accuracy, the test does not require gynecological collection, as testing urine samples with NAATs is at least as sensitive as testing endocervical samples, vaginal samples (whether collected by physicians or self-collected), or urethral samples in clinical settings. Another advantage is that the same sample can be used to test for both chlamydia and gonorrhea. Since there are no studies on optimal screening intervals, a reasonable approach would be to retest patients who remain at risk since their last negative test result.

Intimate partner violence: The USPSTF recommends that clinicians screen women of reproductive age for intimate partner violence (IPV) and provide or refer those who test positive to support services (grade B) [51]. The term "intimate partner violence" refers to physical, sexual, or psychological violence (including coercive tactics, such as limiting access to financial resources) or stalking by a romantic or sexual partner, including spouses, boyfriends, girlfriends, and casual encounters.

The following instruments accurately detect intimate partner violence (IPV) in the past year among adult women: Humiliation, Afraid, Rape, Kick (HARK); Hurt, Insult, Threaten, Scream (HITS); Extended Hurt, Insult, Threaten, Scream (E-HITS); Partner Violence Screen (PVS); and Woman Abuse Screening Tool (WAST). These instruments should be used during the interview, ideally with the woman separated from her partner. Those with positive results should be referred to support services, and in cases of serious violence risk, they should be advised to seek legal or police assistance. Some jurisdictions require doctors to report abuse to law enforcement, particularly injuries resulting from knives or other weapons.

Folic acid supplementation: The USPSTF recommends that all women who plan to become pregnant or may become pregnant take a daily supplement containing 0.4 to 0.8 mg (400 to 800 mcg) of folic acid (grade A) [52]. The use of folic acid aims to minimize the occurrence of neural tube defects. Since the neural plate completes its formation and closure early in pregnancy (usually 26 to 28 days after fertilization), the critical period for folic acid supplementation begins at least one month before conception and continues through the first two to three months of pregnancy. To obtain the full benefits of supplementation, doctors should advise all individuals who plan to become pregnant or may become pregnant to take folic acid daily. Women with a personal, partner, or family history of neural tube defects, malabsorption due to bariatric procedures, diabetes, obesity, the use of certain anticonvulsant medications, or genetic mutations in folate-related enzymes are at increased risk.

Cervical cancer: The USPSTF recommends screening for all asymptomatic women aged 21 to 65 years, regardless of their sexual history (grade A) [53]. The CTFPHC recommends screening for women aged 30 to 69 years (strong recommendation with high-quality evidence). For women aged 25 to 29, the evidence is weaker [54]. Both organizations recommend cervical cytology every three years for women with a normal previous exam. Alternatively, the USPSTF recommends screening with the high-risk human papillomavirus (hrHPV) test alone or in combination with cytology every five years.

Breast cancer: The USPSTF recommends screening for women at general risk between the ages of 40 and 74 (grade B) [55]. Currently, the CTFPHC is revising its guidance, but the draft recommendation suggests conditional screening for women aged 40 to 74 who agree to the procedure after a shared and informed decision-making process (evidence of very low certainty) [56]. Both organizations recommend screening by mammography, with an interval of every two years (USPSTF) or every two to three years (CTFPHC).

BRCA-related cancer: The USPSTF recommends screening for mutations in the breast cancer susceptibility genes BRCA1 and BRCA2 in women considered at risk (grade B) [57]. This includes women with a personal or family history of breast, ovarian, fallopian tube, or peritoneal cancer, or a family history of mutations in BRCA1 or BRCA2.

Screening should occur only once through a detailed interview, in conjunction with various risk analysis tools such as the Ontario Family History Assessment Tool, the Manchester Scoring System, and the Referral Screening Tool. If screening with any of these instruments is positive, genetic counseling and, if indicated, genetic testing for BRCA1 and BRCA2 mutations are recommended. Genetic counseling involves a detailed analysis of the risk and possible consequences of BRCA1 or BRCA2 mutations, along with a thorough kinship analysis. Finally, if a carrier of one of these mutations is identified, potential measures may include intensive surveillance, risk-reducing medications, and mastectomy and/or oophorectomy to reduce risk.

Medication to reduce the risk of breast cancer: The USPSTF recommends that physicians offer to prescribe risk-reducing medications, such as tamoxifen, raloxifene, or aromatase inhibitors, to women at increased risk of breast cancer and low risk of adverse effects from the medication (grade B) [58]. Several risk assessment tools are used to estimate the risk of developing breast cancer in the next five years [59]. Women with a risk of 3% or greater are more likely to benefit from the medications, with the benefits outweighing the risks. The risk is also increased by having first-degree relatives who developed the disease, especially if before the age of 50, the presence of atypical ductal or lobular hyperplasia, or lobular carcinoma in situ in a previous biopsy. Women with documented pathogenic mutations in the breast cancer susceptibility genes BRCA1/2 and women with a history of thoracic radiation therapy are at especially high risk of breast cancer. Although there is no evidence on the optimal interval to reassess risk, it would be reasonable to repeat the risk assessment if there is a significant change in breast cancer risk factors.

When the use of medications is indicated, tamoxifen is the preferred option for reducing the risk of primary breast cancer in premenopausal women, while raloxifene and aromatase inhibitors are preferred for postmenopausal women. When considering prescribing breast cancer risk-reducing medications, the potential benefits of risk reduction must be balanced against the potential harms of adverse effects. Tamoxifen and raloxifene are associated with an increased risk of venous thromboembolic events and vasomotor symptoms. Tamoxifen also increases the risk of endometrial cancer and cataracts. The harms of aromatase inhibitors include vasomotor symptoms, gastrointestinal symptoms, musculoskeletal pain, and possibly an increased risk of cardiovascular events. Younger women with no risk factors for CVD are less likely to experience a cardiovascular event with aromatase inhibitor treatment. Unlike tamoxifen and raloxifene, aromatase inhibitors do not reduce, and may even increase, the risk of fractures.

Osteoporosis screening to prevent fractures: The USPSTF recommends osteoporosis screening to prevent fractures in females over 65 years of age, and in postmenopausal women under 65 years of age who are at increased risk of osteoporotic fracture based on a clinical risk estimation instrument (grade B) [60]. In the latter group, the presence of one or more of the following factors (in addition to the postmenopausal state), low body weight, a parental history of hip fracture, smoking, and excessive alcohol consumption, indicates the need to use a risk assessment tool to determine whether screening is warranted.

The USPSTF cites tools for osteoporosis risk assessment (e.g., ORAI - Osteoporosis Risk Assessment Instrument and OST - Osteoporosis Self-Assessment Tool [61]), as well as tools for fracture risk assessment (such as the FRAX - Fracture Risk Assessment Tool [62], FRC - Fracture Risk Calculator, and the Garvan Fracture Risk Calculator). For osteoporosis screening, the USPSTF identifies the most common method as the measurement of bone mineral density (BMD) using dual-energy X-ray absorptiometry (DXA) in the central areas (e.g., total hip, femoral neck, or lumbar spine). The test can be performed alone or in conjunction with one of the fracture risk assessment tools.

The USPSTF concludes that the current evidence is insufficient to assess the balance between the benefits and harms of osteoporosis screening to prevent osteoporotic fractures in men. Transgender men and transgender women who have not undergone hormonal treatment associated with transitioning likely have the same risks as their birth sex, but it is recommended that they consult with their physician to determine which recommendation best applies to them. The CTFPHC recommends screening women over 65 years of age after an informed and shared decision, using the FRAX [63]. If, after the informed discussion, the use of preventive medication is considered, BMD measurement in the femoral neck is indicated, and risk reassessment should include adding the BMD T-score to the FRAX (conditional recommendation, low-certainty evidence) [64]. The interval between screenings remains undetermined, and although there is limited evidence, it did not show any benefit from repeating the first densitometry between four and eight years.

Specific Recommendations for Adult Males

Abdominal aortic aneurysm: The CTFPHC recommends abdominal aortic aneurysm screening for men aged 65 to 80 years (weak recommendation with moderate quality of evidence) [65], whereas the USPSTF recommends screening for those aged 65 to 75 years with a history of smoking (grade B) [66]. Screening should be done only once using duplex ultrasound and an abdominal scan. For patients with reduced life expectancy or those unable to undergo treatment, screening should not be offered.

Table 1 provides a summary of the recommendations, the target group, and, when applicable, the frequency of repeat measurements.

**Table 1:** Summary of procedures to be established in an evidence-based checkup Insufficient evidence: Current evidence is insufficient to establish optimal time intervals or strategies for repeating the screening. Pragmatic: Although evidence on the need for retesting is insufficient, a pragmatic approach is to retest annually for those who remain at risk or have the condition of interest. *At the time of writing, this was an active recommendation from the USPSTF, but it was in the process of being updated.

Sex	Subgroup	Initial conduct	Condition	Periodicity
Both	All	Screening	Smoking	All consultations
Both	All	Screening	Harmful use of alcohol*	Insufficient evidence
Both	All	Screening	Harmful use of illicit drugs	Insufficient evidence
Both	All	Screening	Depression and suicide risk	Insufficient evidence
Both	All	Screening	Hypertension	All consultations
Both	Obese people	Counseling	Weight loss in obese people*	Pragmatic
Both	15-65 years	Screening	HIV*	Pragmatic
Both	18-64 years	Screening	Anxiety disorders	Pragmatic
Both	18-79 years	Screening	Hepatitis C	Pragmatic
Both	45-75 years old	Screening	Colorectal cancer	(see text)
Both	Older than 65 years old	Counseling	Risk of falls in the elderly	All consultations
Both	6 months-24 years old	Counseling	Skin cancer prevention	All consultations
Both	35-70 years old who are overweight or obese	Screening	Diabetes and prediabetes	Triennial
Both	40-75 years old with cardiovascular risk	Drug prescription	Statin use	All consultations
Both	50-80 years >20 pack/year and <15 years of abstinence	Screening	Lung cancer	Annual
Both	Cardiovascular risk	Counseling	Healthy habits (diet and exercise)*	Pragmatic
Both	Infectious risk/transmission	Screening	Hepatitis B	Pragmatic
Both	Infectious risk/transmission	Screening	Syphilis	3 months/1 year
Both	Infectious risk/transmission	Screening	Latent tuberculosis	Pragmatic
Both	Infectious risk/transmission	Counseling	Sexual behavior	Pragmatic
Both	Specific risk	Drug prescription	HIV pre-exposure prophylaxis	Pragmatic
Female	Sexually active up to 24 years	Screening	Chlamydia and gonorrhea infection	Pragmatic
Female	Reproductive age	Screening	Intimate partner violence*	Insufficient evidence
Female	Reproductive age	Drug prescription	Prevention of neural tube defects	All consultations
Female	21-65 years old	Screening	Cervical cancer*	Triennial
Female	40-70 years old	Screening	Breast cancer	Biennial
Female	>65 years old	Screening	Osteoporosis to prevent fractures	Insufficient evidence
Female	Genetic risk	Screening	BRCA-related cancer*	Once in a lifetime
Female	>35 years old at increased risk for breast cancer	Drug prescription	Reduce risk of breast cancer*	Pragmatic
Male	65-80 years	Screening	Abdominal aortic aneurysm	Once in a lifetime

## Conclusions

The practice of subjecting patients to broad test profiles during an annual checkup continues despite the lack of evidence supporting its benefits and even the possibility that it may be harmful. In this paper, we describe the practices with the greatest potential for usefulness, based on updated scientific evidence, as well as the instruments for implementing them and the frequency of reassessment, if indicated. The measures described here are continually updated, and periodic monitoring is recommended.

By summarizing and focusing on the recommended measures, we hope to contribute to safer medical practices, especially for asymptomatic patients, who visit their physicians in the hope of preserving their health and achieving a longer, more productive life. It is important to remember that these recommendations are constantly evolving, and several are in the process of being reevaluated.
